# Bibliography

**Published:** 1918-10

**Authors:** 


### BIBLIOGRAPHY

### Oral Diseases and Malformations.

The third edition of Dr. G. V. I. Brown's well known work on Oral Surgery has just come from the press of Lea and Febiger. It is a large work of more than 700 pages with more than 500 illustrations. It is printed in the usual style of excellence which characterizes the books of this house. There is little that can be criticised in the mechanical construction of the book. In fact, the work of the printer has added materially to the text.

The new material in this edition is largely made up of the methods that have been developed out of the war experience of the past three years. Owing to the large number of head fractures this phase of surgery has made wonderful progress and has required many new procedures in the way of restorative operations, appliances and treatments. It is almost too early in our army experience to dogmatize in regard to the treatment of such wounds but Dr. Brown has handled his new material with skill and conservation. It is not practicable in a brief notice of this kind to offer an extensive criticism of a book of such dimensions even were it necessary. Dr. Brown is one of our most industrious and painstaking oral surgeons and his writings have always been characterized for thorough and scientific treatment. This work shows no signs of relaxation in the ideals that have heretofore characterized his work.

This book, like Garretson's and Brophy's, is so comprehensive in its contents that it must necessarily make its strongest appeal to the general surgeon or the oral surgeon who undertakes the treatment of all forms of head and face surgery and the diseases of that region. It is a work more for the specialist or the practitioner of oral surgery, rather than a text book for dental students who need only the fundamentals. To illustrate our meaning, it will be somewhat disappointing to the dentist who wants to get fuller instruction in dental surgery, such as extracting impacted teeth, or in the removal of sections of tooth roots, or the necrotic results of alveolar abscesses, etc.—these conditions that every general practitioner must treat occasionally in a surgical way. Undoubtedly it would be difficult if not impracticable to compile a book on this subject that would draw distinct lines between strictly dental surgery and oral or head surgery; that would be sufficiently comprehensive to satisfy all the requirements of the dentist and at the same time have the respect of the general surgeon. No author as yet has had the skill to do this, but perhaps when we shall have accumulated sufficient data it may be accomplished. The dental and oral surgeons are certainly under obligation to both Dr. Brown and the publishers for a most valuable contribution to our literature on the surgical treatment of the diseases of the head and mouth.

